# Selenite-Doped β‑TCP Powders with Improved Biological Properties: Microwave-Assisted Synthesis and Detailed Characterization

**DOI:** 10.1021/acsomega.6c03281

**Published:** 2026-06-22

**Authors:** Azade Yelten Coşkun, Defne Ecem Gür, Gizem Karaaslan, Batur Ercan

**Affiliations:** † Department of Metallurgical and Materials Engineering, Istanbul University-Cerrahpasa, Istanbul 34320, Türkiye; ‡ Department of Metallurgical and Materials Engineering, Middle East Technical University, Ankara 06800, Türkiye; § Biomedical Engineering Program, Middle East Technical University, Ankara 06800, Türkiye; ∥ BIOMATEN, METU Center of Excellence in Biomaterials and Tissue Engineering, Ankara 06800, Türkiye

## Abstract

β-Tricalcium phosphate (β-TCP, Ca_3_(PO_4_)_2_) is a widely used bioresorbable ceramic in orthopedic applications, particularly as a bone graft material and as a coating on bioinert metallic implants. Its bioactivity, resorption kinetics, and biocompatibility account for its extensive clinical use, motivating efforts to further enhance its performance. The incorporation of various ions into the β-TCP lattice has been widely explored to tailor its structural, physicochemical, and biological properties. Among these, selenium (Se) is an essential trace element that enhances osteoblast activity and supports bone regeneration. To achieve this, microwave-assisted synthesis was employed for its advantages, including rapid reaction kinetics, homogeneous heating, limited grain growth, and efficient ion incorporation. In this study, submicron-sized selenite (SeO_3_^2–^)-doped β-TCP (Se-β-TCP) powders were successfully synthesized using this technique and characterized in detail. Structural analyses confirmed the incorporation of SeO_3_^2–^ into the β-TCP lattice, as evidenced by a reduction in the c parameter from 37.372 Å to 37.242 Å and the emergence of a Se–O vibration band at 904–806 cm^–1^, in the infrared spectra. In vitro results showed that MC3T3-E1 preosteoblasts cultured with Se-β-TCP proliferated for up to 5 days, while the powders inhibited colony growth of *Staphylococcus aureus*, *Staphylococcus epidermidis*, and *Escherichia coli*. These findings demonstrate that Se-β-TCP synthesized via microwave-assisted processing is both cytocompatible and antibacterial, highlighting its potential for orthopedic applications.

## Graphical abstract

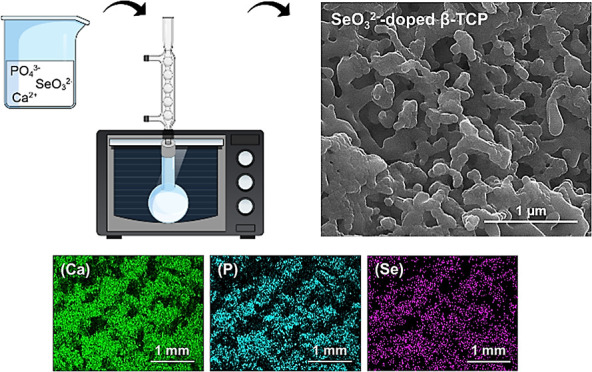

## 1 Introduction

Tricalcium phosphate (β-TCP, β-Ca_3_(PO_4_)_2_) is a well-known bioceramic recognized for its strong ability to promote rapid bone regeneration. It belongs to the resorbable members of the calcium phosphate (CaP) family and possesses a Ca/P molar ratio of 1.5.^1^ Due to its favorable bioactivity and resorption behavior, β-TCP has been utilized in various forms, such as powders, putties, and coatings, particularly in applications requiring a rapid material–tissue response.^2^ Beyond its intrinsic bioactivity, the biological performance of β-TCP can be further tailored through ion incorporation, such as Ce^3+^, Sr^2+^, Ag^+^, Cu^2+^, and Zn^2+^, to impart additional functionalities, including enhanced osteogenic response and antibacterial activity.^1,3^ This antibacterial functionalization is especially relevant for mitigating implant-associated infections.

Despite its bioactivity and biocompatibility, β-TCP, like many implant materials, remains susceptible to bacterial colonization. Once bacteria adhere to an implant surface, they can form a biofilm, which is a self-produced polysaccharide matrix that renders them up to 1000 times more resistant to antibiotics than their planktonic counterparts.^4^ Such infections can ultimately lead to implant failure, tissue necrosis, or, in severe cases, amputation. With the increasing prevalence of antibiotic-resistant strains, such as methicillin-resistant *Staphylococcus aureus* (MRSA), there is an urgent need to develop implant materials that inherently resist bacterial colonization. For β-TCP, one promising approach involves incorporating selenium, either in the elemental form^5^ or as selenite anions,^6^ into its crystal structure. Selenium is an essential trace element in humans, integrated into the amino acid selenocysteine (Sec), which is critical for the synthesis of various selenoproteins involved in antioxidant and enzymatic activity.^7^ In our previous work, we demonstrated that amorphous calcium–magnesium carbonate nanoparticles doped with selenite exhibited potent antibacterial activity, significantly inhibiting the growth of both Gram-positive (*S. aureus* and *Staphylococcus epidermidis*) and Gram-negative (*Escherichia coli* and *Pseudomonas aeruginosa*) bacteria within 24 h.^8^ Building on these findings, selenite (SeO_3_^2–^) has emerged as a promising dopant for CaP-based structures such as hydroxyapatite^9,10^ and β-TCP, offering a route to introduce antibacterial functionality while retaining their bioactive properties. Accordingly, the synthesis technique for SeO_3_^2^- doped β-TCP is critical, as it governs particle size, morphology, structure, and functional performance.^11,12^

Several wet-chemical approaches, including chemical precipitation, hydrothermal synthesis, and sol–gel processing, have been used to synthesize both nondoped and ion-doped β-TCP.^13^ Among these, microwave-assisted synthesis has attracted particular interest due to its rapid reaction kinetics, energy efficiency, and environmental sustainability. This technique enables the formation of highly uniform β-TCP particles with controlled size and morphology while minimizing grain growth through markedly reduced reaction times.^11,12,14^ Unlike conventional heating, where thermal energy is transferred gradually from the surface inward, microwave irradiation generates heat volumetrically within the material by directly interacting with the reactants. During β-TCP synthesis, the interaction between calcium and phosphorus precursors under microwave irradiation enhances molecular vibrations, causing the reaction medium to behave like a micromagnet. This intensified molecular motion, coupled with efficient absorption of electromagnetic energy, results in rapid and homogeneous heating throughout the system. Consequently, microwave-assisted synthesis effectively suppresses grain coarsening and promotes a narrow, uniform particle size distribution.^15,16^

Motivated by the growing interest in antibacterial ion-doped β-TCP, this study focuses on incorporating selenite (SeO_3_^2–^) as a functional dopant to mitigate bacterial infections in orthopedic applications. Among widely used antibacterial agents, certain Se-containing species have been reported to exhibit anticancer activity through the induction of apoptosis in cancer cells.^9,10,17,18^ In this context, our preliminary research on the microwave-assisted synthesis of SeO_3_^2–^-doped β-TCP (Se-β-TCP) powder^19,20^ was significantly expanded with additional characterization techniques in the present study, in which extended microwave irradiation (15 min) was applied to determine whether the synthesized phase retains its stability and whether secondary phases form. Additionally, it was intended to allow the dopant ion to be incorporated into the crystal structure and disperse more homogeneously during longer microwave irradiation.^21,22^ The physical, chemical, and thermal properties of the achieved powders were thoroughly analyzed using X-ray diffraction (XRD), Fourier-transform infrared (FT-IR) spectroscopy, particle size analysis, scanning electron microscopy (SEM), energy-dispersive spectroscopy (EDS), transmission electron microscopy (TEM), and differential scanning calorimetry/thermogravimetric analysis (DSC-TG). In vitro bioactivity of the samples was evaluated by immersing them in 1X simulated body fluid (SBF). The antibacterial performance of the synthesized powder was assessed against both Gram-positive strains (*S. aureus* and *S. epidermidis*) and a Gram-negative strain (*E. coli*). Furthermore, the cytocompatibility of Se-β-TCP was assessed through an osteoblast proliferation assay to compare cellular growth and evaluate its suitability for orthopedic applications.

## 2 Materials and Methods

### 2.1 Preparation of the Powders

Undoped (β-TCP) and Se-β-TCP powders were synthesized using the microwave-assisted method described in our initial studies,^19,20^ with adjustments to the microwave reaction time. Briefly, a modified household microwave oven was used to create a cost-effective, practical setup consisting of the microwave unit, a one-neck glass flask, and a reflux condenser connected via an adaptor. Aqueous calcium (Ca) and phosphorus (P) source solutions were prepared using calcium nitrate tetrahydrate (Ca­(NO_3_)_2_·4H_2_O, Sigma-Aldrich, 99%) and diammonium hydrogen phosphate ((NH_4_)_2_HPO_4_, Sigma-Aldrich, ≥98%), respectively. The Ca/P molar ratio was adjusted to 1.5 to match the stoichiometry of the β-TCP phase for both undoped and ion-doped samples. For the Se-β-TCP powders, sodium selenite (Na_2_SeO_3_, Sigma, 99%) was added as 1% mole of its molecular weight, corresponding to 6% moles in the system, enabling the partial substitution of SeO_3_^2–^ ions for PO_4_^3–^ ions. It was intended to compensate for the possible limited incorporation of dopant ions^10^ due to ion losses that may occur during process stages, such as rinsing. After rapidly adding the phosphorus solution to the calcium solution, the mixture was stirred briefly. Ammonia solution (Isolab, NH_4_OH, 25%) was then added to adjust the pH to 8, while maintaining vigorous stirring. The mixture was transferred to the one-neck flask, placed inside the microwave oven, and irradiated at 800 W for 15 min. After microwave exposure, the flask was removed from the oven and allowed to cool to room temperature. The resulting slurry was centrifuged at 5000 rpm for 1 min to separate the precipitate from the supernatant. The collected precipitate was rinsed multiple times with deionized water to remove residual contaminants and then dried overnight at 80 °C. The dried powder was ground manually with a mortar and pestle and then calcined at 900 °C for 1 h at a heating rate of 15 °C/min. The overall synthesis procedure is illustrated in Figure 1.

**Figure 1:**
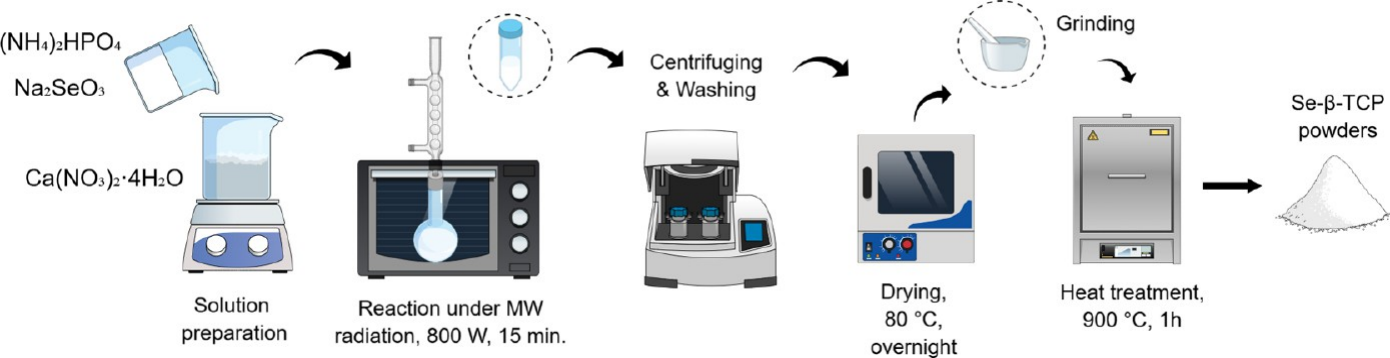
Experimental procedure used to synthesize the Se-β-TCP powders. (This figure is created by using scientific illustration elements from mindthegraph.com).

### 2.2 Characterization of the Powders

The calcined powders were characterized to evaluate their physical, chemical, and thermal properties. Phase identification was performed using XRD on a Rigaku D/Max-2200 diffractometer over a 2θ range of 10° to 80° with a scanning rate of 2°/min. The lattice parameters (a and c) of the (calcined) β-TCP and Se-β-TCP powders were calculated using eq 1, which is applicable to hexagonal crystal structures, where *h*, *k*, and *l* represent the Miller indices. Although β-TCP crystallizes in the rhombohedral space group *R*3*c*,^23,24^ its lattice can be conveniently described using the hexagonal setting. Therefore, the lattice parameters were calculated using the hexagonal approximation to simplify the unit-cell representation.^25,26^ The interplanar spacing (*d*_*hkl*_) values were determined according to Bragg’s Law (eq 2), where λ denotes the X-ray wavelength (Å) and θ is the diffraction angle (°).

1 $$d_{hkl}=\frac{a}{\sqrt{\frac{4}{3}\left(h^{2}+hk+k^{2}\right)+\frac{a^{2}}{c^{2}}l^{2}}}$$

2 n×λ=2d×sin⁡θ

The molecular bonding characteristics of the samples were examined using Fourier-transform infrared (FT-IR, Jasco 4700) spectroscopy, equipped with an attenuated total reflectance accessory. The morphology of the powders was observed using scanning electron microscopy (SEM; FEI Nova Nano 430 and Hitachi SU5000) after coating the samples with a thin layer of gold to provide electrical conductivity (Quorum SC7640 High-Resolution Sputter Coater). Elemental mapping was performed using energy-dispersive spectroscopy (EDS; Hitachi SU3500 equipped with an Oxford Instruments X-act) integrated with the SEM. The internal structure of the particles was investigated using TEM (FEI Tecnai F30). Particle size distributions were determined using dynamic light scattering (DLS, Malvern Zetasizer Nano ZS90) employing distilled water as the dispersion medium and a refractive index of 1.6. The thermal behavior of the as-precipitated powders was investigated up to 1000 °C using a differential scanning calorimetry/thermogravimetric analyzer (DSC-TG, Discovery SDT 650) under dry air, with a heating rate of 10 °C/min and a gas flow rate of 100 mL/min. The presence of Se content of the Se-β-TCP powder was measured by inductively coupled plasma mass spectrometry (ICP–MS, PerkinElmer DRC II). The degradation behavior of powders was investigated by immersing the samples in phosphate-buffered saline (1X PBS solution, pH: 7.4) at 37 °C for up to 21 days. The pH of the PBS solution was measured on days 1, 2, 3, 4, 7, 14, and 21. The in vitro bioactivity of the powders was assessed by immersing the samples in 1X SBF solution, prepared according to Kokubo’s protocol,^27^ for 7 and 21 days at 37 °C. The CaP precipitates formed on the sample surfaces over time were characterized by XRD, FT-IR, and SEM.

### 2.3 Investigation of Bone Cell Proliferation

MC3T3-E1 preosteoblast cells (ATCC CRL-2593) were cultured under standard cell culture conditions (37 °C, 5% CO_2_) in Minimum Essential Medium α supplemented with 10% fetal bovine serum, 1% l-glutamine, and 1% penicillin/streptomycin. Prior to biological experiments, the powders were sterilized by immersion in 70% (v/v) ethanol for 30 min and then washed in 1X PBS for 30 min. The washed samples were air-dried and exposed to UV light for 1 h.

For osteoblast proliferation experiments, sterile powders were extracted in the growth medium at 0.01 g/mL and incubated at 37 °C for 72 h, in accordance with ISO 10993-5. Cells were seeded at a density of 10,000 cells/well. After 24 h, the growth medium was aspirated and replaced with the extract solutions. Cell metabolic activity was evaluated on days 1, 3, and 5 using a 3-(4,5-dimethylthiazol-2-yl)-2,5-diphenyltetrazolium bromide (MTT) assay. At each time point, extracts were aspirated, and cells were rinsed with 1X PBS. Subsequently, 100 μL of MTT solution was added to each well and incubated for 4 h at 37 °C in 5% CO_2_ to allow the MTT solution to react with metabolic products. After incubation, 100 μL of 2-propanol was added to dissolve the resulting formazan crystals, and the absorbance was measured at 560 nm using a spectrophotometer. The total cell count in each well was calculated using a standard absorbance–cell number graph obtained at the beginning of the experiment.

For cell morphology analysis, cells were cultured in the extract solutions for 3 days, as described above. After incubation, the medium was removed, and the cells were rinsed with 1× PBS. They were then fixed with 4% paraformaldehyde, rinsed again, and dehydrated through a graded ethanol series (30%, 50%, 70%, 90%, 95%, and 100% v/v). Hexamethyldisilazane was subsequently applied, and the samples were left to dry for 24 h. Before SEM imaging, the cells were coated with a thin gold layer using a sputter coater to ensure electrical conductivity.

### 2.4 Antibacterial Properties

Before antibacterial testing, the powders were sterilized as described in Section 2.3. Sterilized powders were extracted in 1× PBS at 0.01 g/mL and incubated at 37 °C for 72 h. Antibacterial activity was evaluated against the following bacterial strains: Gram-positive *S. aureus* (ATCC 25923) and *S. epidermidis* (ATCC 35984) and Gram-negative *E. coli* (ATCC 10536). Standard bacterial culture protocols were followed. Briefly, bacteria were first streaked onto tryptic soy agar (TSA) plates and incubated at 37 °C for 24 h to obtain isolated colonies. A single colony from each strain was then transferred to tryptic soy broth (TSB) and cultured in a shaking incubator at 37 °C with agitation at 200 rpm for 4 h. The optical density of the cultures was measured at 600 nm and adjusted to 0.1, corresponding to approximately 1.5 × 10^8^ colony-forming units (CFU)/mL, based on the 0.5 McFarland scale.^28^ The bacterial suspensions were then further diluted in TSB to obtain a final concentration of approximately 10^6^ CFU/mL. Equal volumes (100 μL) of bacterial suspensions and powder extracts were then mixed in 96-well plates and incubated at 37 °C for 24 h. After incubation, the contents of each well were serially diluted in sterile 1X PSB and spread onto TSA plates for colony enumeration. The plates were incubated again at 37 °C for 24 h, after which the number of bacterial colonies was counted to determine the viable cell count.

### 2.5 Statistical Analysis

All biological experiments were performed in triplicate, with each replicate comprising 4 samples. Data are expressed as the mean ± standard deviation. Statistical analysis was performed using one-way analysis of variance (ANOVA), followed by Tukey’s post hoc test to evaluate differences among groups. A *p*-value <0.05 was considered statistically significant.

## 3 Results and Discussion

XRD results of the powders before and after the calcination process are shown in Figure 2a,b. XRD results revealed crystallization of the amorphous CaP-based powders upon the calcination process. Regardless of selenite doping, the as-prepared powders displayed broad peaks, whereas the calcined samples exhibited sharp peaks, indicating an improved crystallinity and particle maturation.

**Figure 2:**
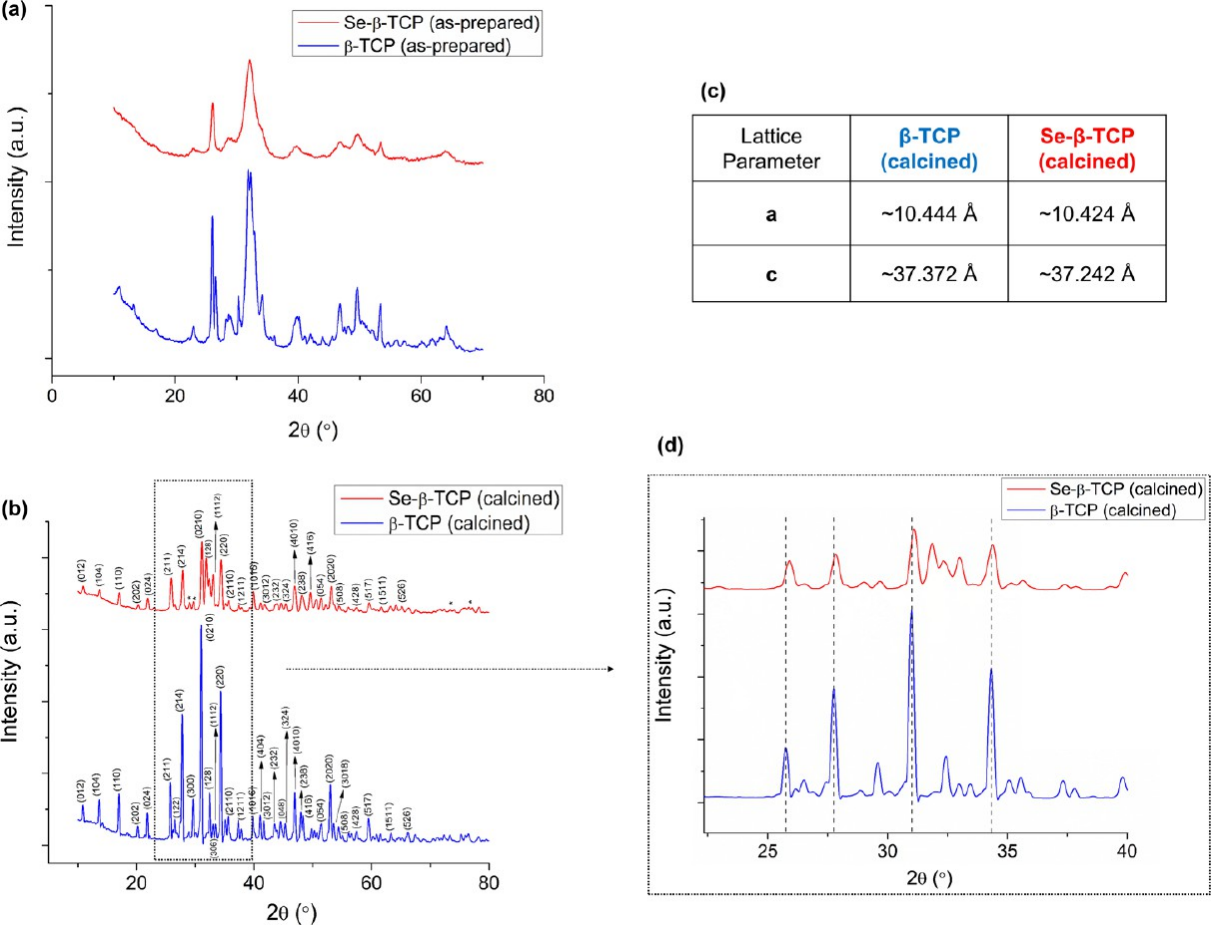
(a,b) XRD patterns of the as-prepared and calcined undoped β-TCP and Se-β-TCP powders and (c) lattice parameters of the calcined undoped β-TCP and Se-β-TCP powders. (d) Peak shifts observed in the spectrum of the powder upon the incorporation of selenite. The calcined undoped β-TCP and Se-β-TCP powders were used in all subsequent analyses.

The XRD patterns of the calcined undoped powder (β-TCP) exhibited only the characteristic diffraction peaks of β-TCP (Ca_3_(PO_4_)_2_, JCPDS Card No. 09-0169),^29^ confirming the absence of secondary or impurity phases. In the Se-β-TCP powders, β-TCP was again identified as the dominant phase, and the main structure remained β-TCP even with a longer microwave irradiation duration (i.e., 15 min), indicating that the extended microwave exposure did not significantly alter the targeted CaP-based phase. Indeed, only weak peaks corresponding to Se_2_O_5_ (JCPDS Card No. 034-1000) were detected as the secondary phase. The reproducibility of both the undoped and Se-β-TCP phases was verified by repeating the synthesis several times with the optimized processing parameters. The Miller indices corresponding to the identified peaks were labeled on the XRD patterns, and the weak peaks associated with Se_2_O_5_ were marked with an asterisk (*). The a and c lattice constants of the undoped β-TCP powders were calculated to be approximately ∼10.444 Å and ∼37.372 Å, respectively. In contrast, those of Se-β-TCP powders were determined to be approximately ∼10.424 Å and ∼37.242 Å (Figure 2c). The lattice constants of the microwave-assisted synthesized β-TCP powder closely match the reported reference values (*a* = *b*: 10.439 Å and *c*: 37.375 Å).^23,24^ While a minor change was observed in the “*a*” lattice constant between β-TCP and Se-β-TCP powders, the reduction in the “*c*” lattice constant was more pronounced. This decrease in the *c* parameter suggests the incorporation of SeO_3_^2–^ ions into the β-TCP lattice, consistent with the peak shifts observed toward higher diffraction angles (Figure 2d). The peak shifts were evaluated comparing the positions of the diffraction peaks at 2θ values of 31.002° (0210) and 34.318° (220) for the undoped β-TCP powders with those at 2θ values of 31.104° (0210) and 34.380° (220) for the Se-β-TCP powders.

Since the ionic radius of SeO_3_^2–^ (0.239 nm) is nearly identical to that of PO_4_^3–^ (0.238 nm), no significant change for the “*a*” lattice constant is expected.^30^ In contrast, the observed contraction of the “*c*” lattice constant is consistent with earlier findings by Kolmas et al.^30^ and Ressler et al.,^10^ who attributed this effect to lattice distortions and vacancy formation resulting from the substitution of PO_4_^3–^ with SeO_3_^2–^ ions. The reduction in the “*c*” lattice constant also affected the sharpness of the XRD peaks. The broader peaks observed for the Se-β-TCP powders suggest reduced crystallinity, which can be attributed to the microstrains arising from lattice distortions caused by the substitution of PO_4_^3–^ groups with SeO_3_^2–^ ions.^31^ In contrast, the β-TCP powders exhibited sharp, well-defined peaks, confirming the presence of a well-crystallized β-TCP phase.^32^

The FT-IR spectra of the β-TCP and Se-β-TCP powders obtained before (Figure 3a) and after (Figure 3b) calcination revealed distinct changes associated with dehydration and crystallization. The disappearance of the broad band observed at approximately 3560–3230 cm^–1^ in the as-prepared β-TCP powder confirmed the removal of adsorbed water introduced during synthesis.^32,33^ This band, attributed to the stretching vibrations of surface hydroxyl groups from physically bound water, was absent after calcination, indicating effective dehydration during heat treatment. In addition, no characteristic sharp absorption bands at approximately 3570 cm^–1^ and 630–635 cm^–1^, which are typical of structural hydroxyl groups (OH^–^) in stoichiometric hydroxyapatite (HA, Ca_10_(PO_4_)_6_(OH)_2_), were detected in either the β-TCP or Se-β-TCP samples.^33–35^ The absence of these HA-related bands confirms that hydroxyapatite did not form as a secondary phase. These FT-IR results are consistent with the XRD findings, which identified β-TCP as the sole phase in the undoped β-TCP powders and the dominant phase in the Se-β-TCP powders.

**Figure 3:**
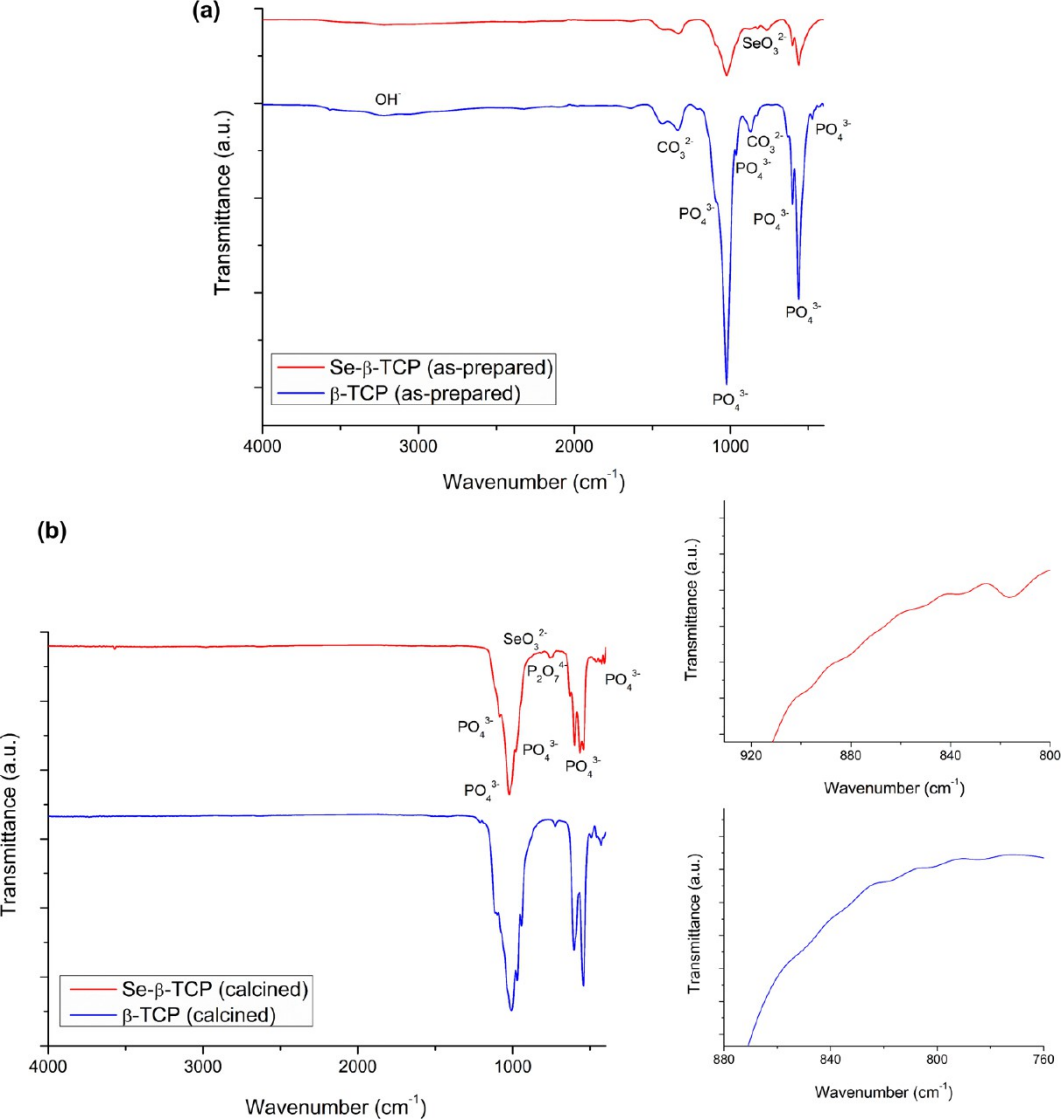
FT-IR results of the (a) as-prepared and (b) calcined undoped β-TCP and Se-β-TCP powders.

The vibrations of the PO_4_^3–^ ions were observed in four modes. The ν_1_ and ν_2_ modes were detected at 961 cm^–1^ and 471 cm^–1^, respectively, for the as-prepared β-TCP powders, whereas the ν_1_ mode appeared at 956 cm^–1^ for the as-prepared Se-β-TCP powders. The ν_3_ mode was observed at 1097 cm^–1^ for the as-prepared β-TCP powders and at 1094 cm^–1^ for the as-prepared Se-β-TCP. In addition, the ν_4_ mode of the PO_4_^3–^ ions was detected at 604 cm^–1^ and 555 cm^–1^ for the as-prepared β-TCP sample and at 596 cm^–1^ and 557 cm^–1^ for the as-prepared Se-β-TCP sample. The bands at 1439 cm^–1^, 1338 cm^–1^, and 866 cm^–1^ observed for the as-prepared β-TCP powders, and those at 1434 cm^–1^ and 1328 cm^–1^ for the as-prepared Se-β-TCP sample, represent the incorporation of the CO_3_^2–^ ions originating from CO_2_ dissolution in the solution under open-air processing conditions.^36^ A broad band was observed between 901 cm^–1^ and 756 cm^–1^ for the Se-β-TCP powders, which was assigned to the Se–O stretching vibrations of the SeO_3_^2–^ ions. Comparison of the FT-IR spectra of the as-prepared undoped β-TCP and as-prepared Se-β-TCP samples shows that the peak at 866 cm^–1^, attributed to the CO_3_^2–^ ions, was less strong in the Se-β-TCP sample due to the overlap of the SeO_3_^2–^ and CO_3_^2–^ vibrational bands.^10,37^

In the calcined undoped β-TCP powder (Figure 3b), the absorption bands at 1100, 1008, 968, and 940 cm^–1^ correspond to the P–O stretching vibrations of the PO_4_^3–^ groups.^10,33,35^ The characteristic sharp peaks at 607 and 546 cm^–1^ are attributed to the bending vibrations of the PO_4_^3–^ ions in the β-TCP phase,^34,38^ and the band at 466 cm^–1^ also represents the bending vibrations of PO_4_^3–^.^39^ These phosphate-related bands were likewise observed in the calcined Se-β-TCP powder at similar wavenumbers, confirming that the primary β-TCP framework remained intact after doping. The shallow peak detected at 760 cm^–1^ in the calcined samples can be attributed to the P–O–P stretching vibrations of the P_2_O_7_^4–^ groups formed during the calcination process at 900 °C.^35^ The incorporation of SeO_3_^2–^ ions was confirmed by the appearance of a distinct band at 904–806 cm^–1^, assigned to the typical vibration modes of the Se–O bonds, and a peak at 816 cm^–1^ associated with the stretching vibrations of SeO_3_^2–^ ions incorporated into the crystal structure of β-TCP.^10,32^

SEM images of the as-prepared and calcined powders are presented in Figure 4. Marked differences were observed between the two in terms of particle size, shape, and agglomeration behavior. The as-prepared powders exhibited a flake-like morphology, whereas the calcined samples displayed a typical sintered structure composed of rounded particles in the submicron range. Notably, both calcined undoped β-TCP and Se-β-TCP particles exhibited a rounded morphology, more pronounced in the selenite-doped sample. The maturation process occurring during calcination at 900 °C for 1 h facilitated crystallization. The average particle sizes of the calcined powders, measured from SEM images using ImageJ, were determined as 331 ± 105 nm for the undoped β-TCP and 133 ± 55 nm for the Se-β-TCP samples. TEM analysis further supported these results, with the mean particle size of the undoped β-TCP measured as 406 ± 123 nm (Figure 5a). Due to the particles’ noticeable tendency toward agglomeration, TEM micrographs (Figure 5a,b) were particularly useful for a more reliable assessment of particle size and morphology. Nevertheless, both SEM and TEM measurements confirmed the formation of submicron-sized particles in the undoped β-TCP and Se-β-TCP powders obtained after 15 min of microwave-assisted synthesis. The TEM images also revealed that the powders exhibited a rounded particle morphology upon calcination.

**Figure 4:**
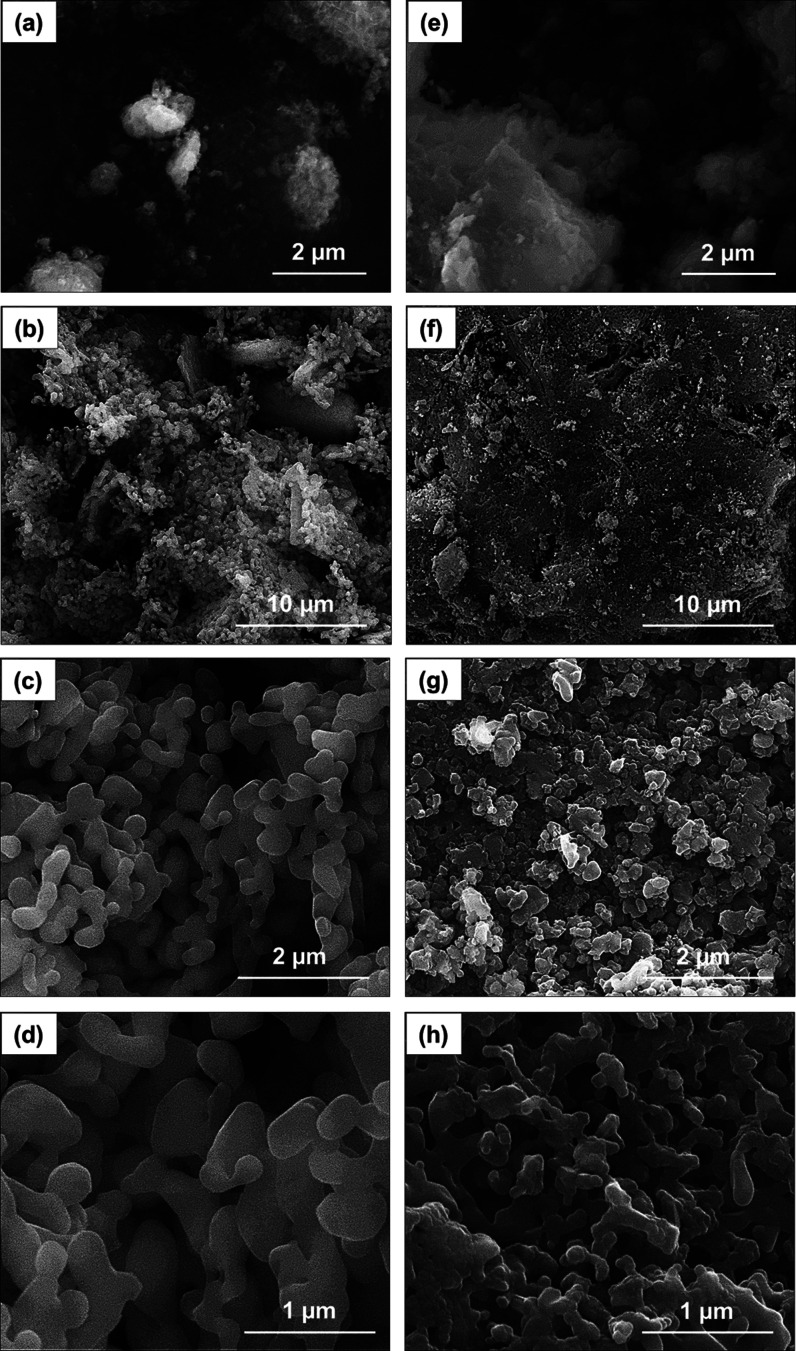
SEM images of the as-prepared (a) undoped β-TCP and (e) Se-β-TCP powders; calcined (b–d) undoped β-TCP and (f–h) Se-β-TCP powders.

**Figure 5:**
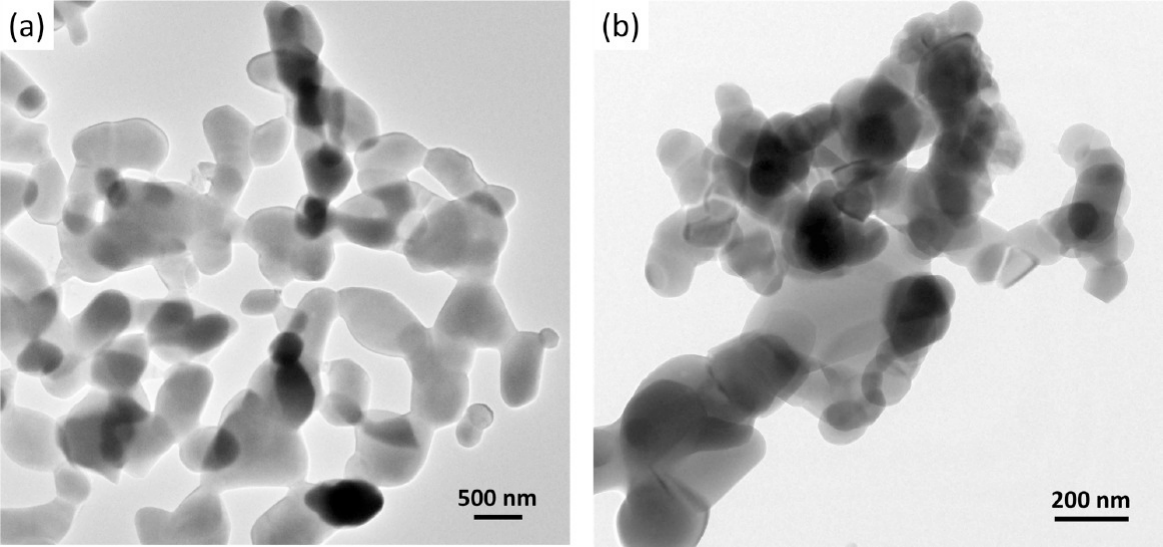
TEM images of the calcined (a) undoped β-TCP and (b) Se-β-TCP powders.

EDS analysis revealed characteristic peaks for Ca, P, and O in the undoped β-TCP powders, while the Se-β-TCP powders additionally exhibited Se peaks, confirming the presence of selenium (Figure 6a). Complementary elemental mapping demonstrated that the detected Se was homogeneously distributed throughout the Se-β-TCP particles, in which the longer microwave irradiation (i.e., 15 min) was thought to be influential on allowing the integration of the dopant ion to the crystal structure (Figure 6b). Nevertheless, Se_2_O_5_ reflections detected by XRD suggest that a fraction of the SeO_3_^2–^ ions was not fully incorporated into the β-TCP lattice, despite their homogeneous distribution observed by elemental mapping. Factors such as the feasibility of the crystal structure for ion substitution, and process steps like rinsing, during which dopant ions may be removed along with residual contaminants,^10,40^ are considered to be effective in explaining the limited but reasonable incorporation of SeO_3_^2–^. Nevertheless, the successful introduction of selenium into the β-TCP powders was further verified by ICP–MS analysis (Table 1).

**Figure 6:**
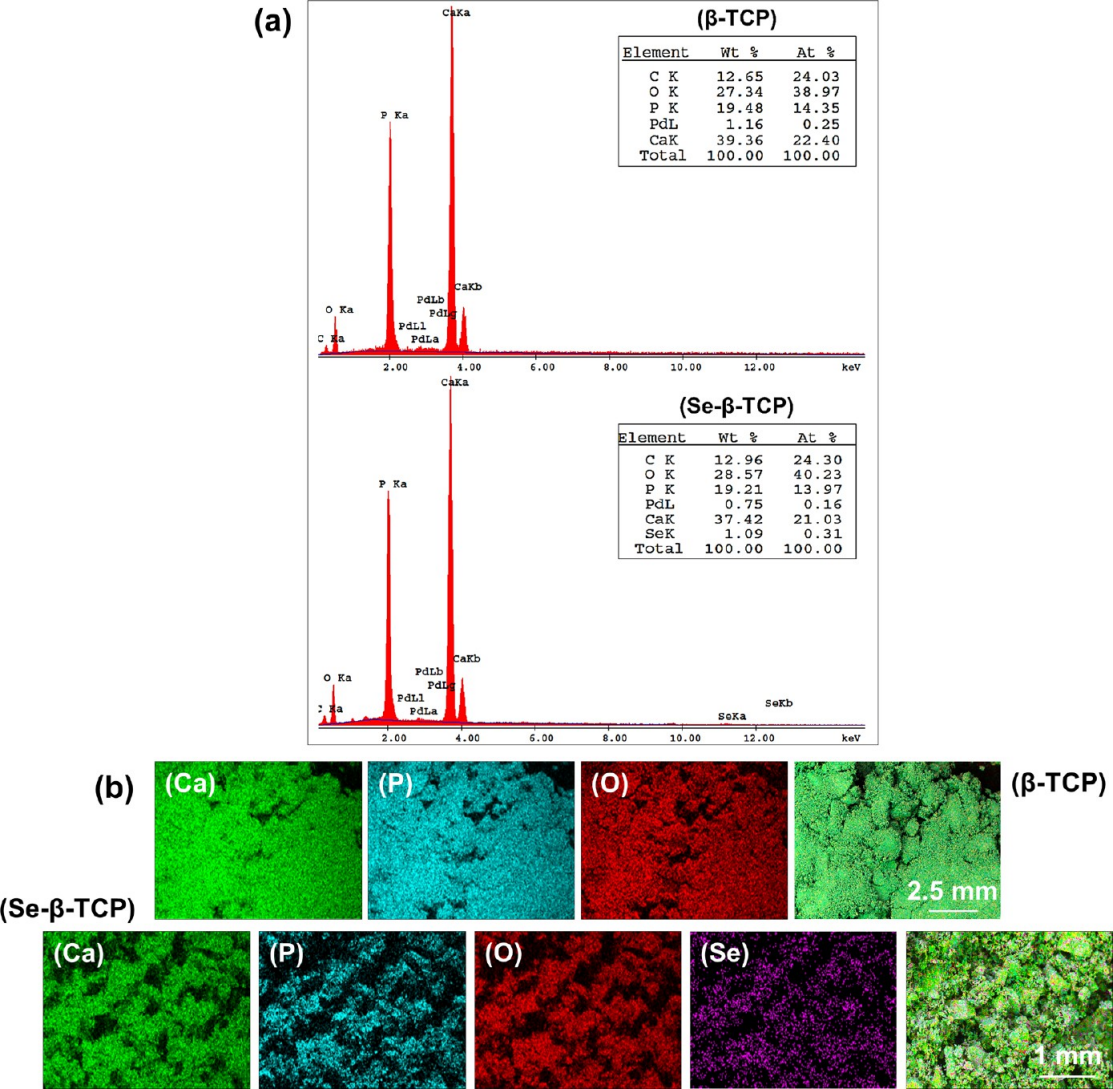
(a) EDS results and (b) elemental mapping of the calcined undoped β-TCP and Se-β-TCP powders.

**Table 1:** ICP–MS Analyses of the Calcined Undoped β-TCP and Se-β-TCP Powders

sample	Ca (wt %)	P (wt %)	Se (wt %)
undoped β-TCP	35 ± 1	22 ± 1	
Se-β-TCP	35 ± 1	20 ± 1	0.60 ± 0.03

The dissolution behavior of the calcined undoped β-TCP and Se-β-TCP powders was evaluated by monitoring changes in the pH of PBS solutions, with the samples immersed at 37 °C for up to 21 days. As shown in Table 2, the pH of the PBS solution containing calcined undoped β-TCP powders increased slightly from 7.47 to 7.71. Consistent with the literature, the undoped β-TCP powders showed dissolution, as evidenced by a slight increase in the pH of the PBS.^41^ The presence of CO_3_^2–^ ions, which are more prominent in the undoped β-TCP samples (Figure 3), is known to increase pH and may have contributed to the higher pH observed for β-TCP powders. In contrast, the PBS solution containing Se-β-TCP powders exhibited a continuous decrease in pH from 7.22 to 6.35, indicating the gradual release of SeO_3_^2–^ ions. Ion incorporation into the structure is known to enhance the dissolution and ion-release behavior of the β-TCP particles. Moreover, doping can alter the crystallinity and structural stability of β-TCP and thereby can increase its susceptibility to degradation.^42–44^ It can be speculated that SeO_3_^2–^ ions released into PBS may react with Ca^2+^ ions to precipitate as CaO_3_Se, thereby contributing to a decrease in pH. Overall, the formation of a more acidic environment appears to be primarily associated with SeO_3_^2–^ doping, which is considered the dominant factor governing the enhanced dissolution behavior of the Se-β-TCP powders.^37,42^

**Table 2:** Changes in the pH of the PBS Solutions during 21 Days of Immersion of Calcined Undoped β-TCP and Se-β-TCP Powders

	pH						
sample	1 d	2 d	3 d	4 d	7 d	14 d	21 d
undoped β-TCP	7.47	7.43	7.51	7.55	7.57	7.77	7.71
Se-β-TCP	7.22	7.00	6.90	6.82	6.60	6.53	6.35

The DSC–TG curves of the as-prepared undoped β-TCP and Se-β-TCP powders recorded up to 1000 °C are presented in Figure 7. The undoped β-TCP powders exhibited an initial weight loss of approximately 8% up to 200 °C, which is attributed to the removal of physically adsorbed water (Figure 7a).^39,45^ A similar trend was also observed for the Se-β-TCP powders, which showed a weight loss of about 12.5% in the same temperature range (Figure 7b). Between 200 and 350 °C, an additional mass loss of ∼7% for the undoped β-TCP and ∼10% for the Se-β-TCP powders was recorded, corresponding to the evaporation of organic impurities, release of chemically bound water, and possible decomposition of ammonia and nitrate remnants.^45^ The greater overall weight loss (∼71%) observed for the Se-β-TCP powders up to 800 °C is attributed to ion incorporation into the structure. Since the substitution of PO_4_^3–^ with SeO_3_^2–^ introduces a charge imbalance, structural modifications and lattice defects occur, as also evidenced by XRD. Consequently, the thermal stability and decomposition behavior of Se-β-TCP become different than that of the undoped β-TCP sample. Owing to the higher degree of structural disorder associated with ion substitution, structural rearrangement in Se-β-TCP appears to occur over a broader temperature range, in contrast to the more ordered crystallization process estimated for undoped β-TCP at approximately 475 °C. Similarly, the shift in the thermal curves of the Se-β-TCP powders may be related to the increased retention of water and volatile species within the modified lattice, resulting from ion incorporation.^46,47^ As the decomposition of carbonates also contributes to the gradual weight loss observed between 200 and 800 °C,^39,48^ the further slight mass loss of approximately 1% for undoped β-TCP and 2% for Se-β-TCP that occurred between 700 and 800 °C is correlated with the departure of the residual carbonate content.^48–50^ Beyond this temperature range, the TG curves remained relatively stable up to 1000 °C, indicating the absence of further significant thermal events within the studied temperature range.^39,45^

**Figure 7:**
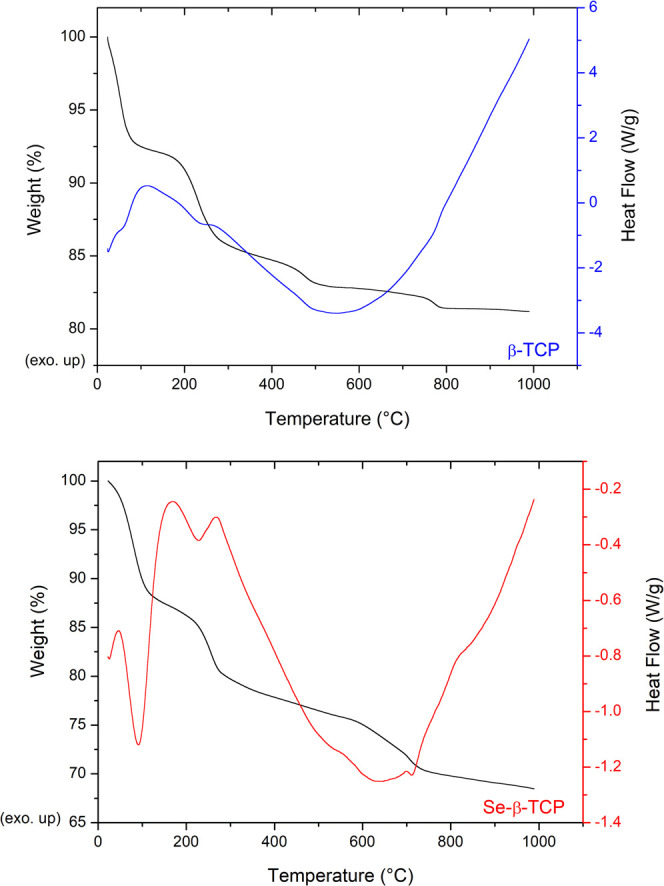
DSC-TG results of the as-prepared undoped β-TCP and Se-β-TCP powders.

FT-IR/XRD and SEM analyses for the in vitro bioactivity tests are presented in Figures 8 and 9, respectively. FT-IR results for both undoped β-TCP and Se-β-TCP samples (Figure 8b) indicate the formation of an apatite-like CaP layer as a function of immersion time in 1X SBF. The characteristic PO_4_^3–^ band at around 541 cm^–1^ for undoped β-TCP and Se-β-TCP powders showed noticeable broadening after 21 days of immersion in SBF. This difference observed in the PO_4_^3–^ characteristic region (labeled as “1” in both FT-IR spectra) reflects the effect of immersion in SBF, resulting in lower crystallinity depending on the formation of initial apatite-like CaP precipitates.^34,36,38^ Consistent with these FT-IR results, additional reflections attributable to calcium phosphate oxide (JCPDS Card No. 01-089-6495) were detected in the XRD patterns of both undoped β-TCP and Se-β-TCP powders (Figure 8a). These reflections, marked with an asterisk (*), appeared at 2θ ranges of 31.78–31.86°, confirming early stage apatite formation.^51,52^

**Figure 8:**
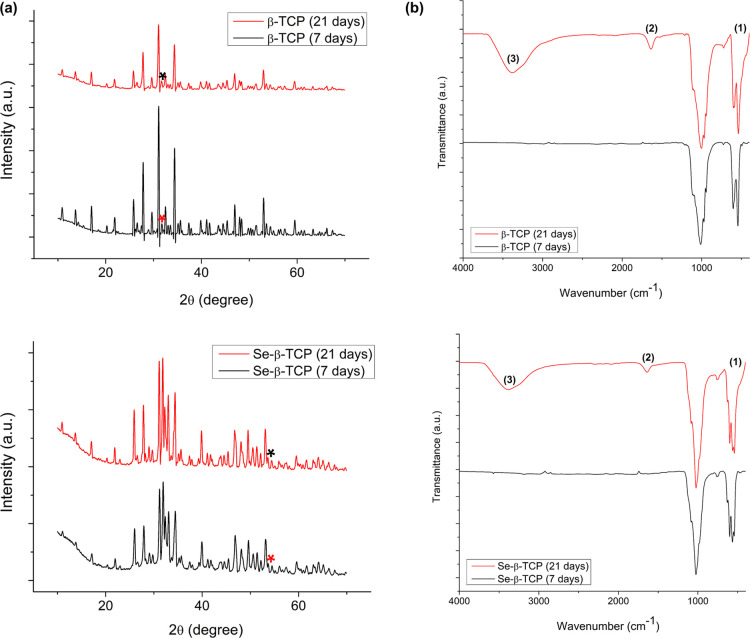
(a) XRD and (b) FT-IR spectra of the calcined undoped β-TCP and Se-β-TCP powders before and after immersion in 1X SBF for up to 21 days. (Ca_10_(PO_4_)_6_O; calcium phosphate oxide, JCPDS Card No. 01-089-6495).

**Figure 9:**
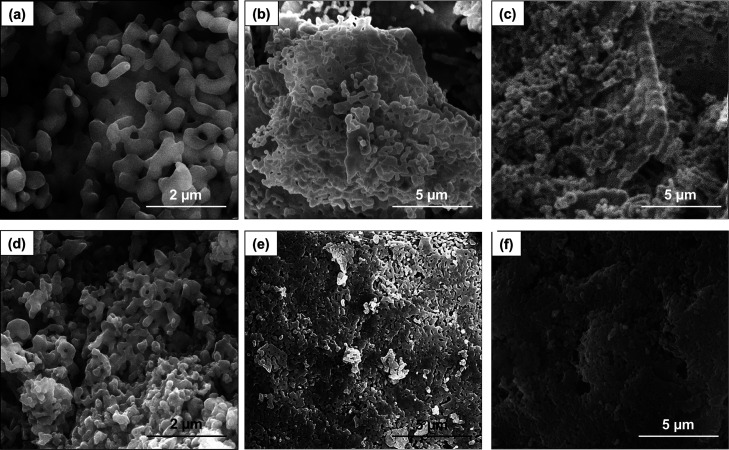
SEM images of (a–c) undoped β-TCP and (d–f) Se-β-TCP powders: (a,d) before immersion; (b,e) after 7 days; and (c,f) after 21 days of immersion in SBF.

The absence of carbonate-related bands in the 1400–1500 cm^–1^ region observed for the as-prepared powder suggests that carbonate incorporation is delayed in the presence of selenite doping, indicating that longer immersion time and a maturation process are required to form carbonated apatite. Because ion doping alters structural stability and crystallinity, it can accelerate dissolution–precipitation processes and thereby facilitate the formation of an initial apatite-like CaP layer, revealing the bioactive character of the samples.^51,53^ Although the introduction of SeO_3_^2–^ ions promoted the precipitation of an initial apatite-like layer compared with undoped β-TCP powders, the incorporation of carbonate ions appears to require more extended maturation due to the reduced crystallinity induced by doping, which hinders carbonate accommodation. Since SeO_3_^2–^ incorporation modifies the crystal structure of β-TCP, it is reasonable to assume that carbonate ions have greater difficulty entering the lattice and that prolonged immersion is therefore necessary for the development of a carbonated apatite layer.^51,54^ Nevertheless, clear bioactivity-related features were observed for the Se-β-TCP powders, as evidenced by both modifications in the conventional vibrational region and increased sharpness of the corresponding FT-IR bands after 7 and 21 days of immersion. In contrast, the band at 1640 cm^–1^ (labeled “2” in both FT-IR spectra) was attributed to surface-adsorbed carbonate species on samples immersed in SBF for 21 days, rather than to the formation of carbonated apatite, which is expected to develop at later stages of immersion.^36^

The broad band at 3380 cm^–1^ (labeled “3” in both FT-IR spectra) was again observed in the samples immersed in 1X SBF for 21 days and was attributed to increased water adsorption and thus to O–H stretching vibrations, as expected. A higher intensity of this band suggests greater surface hydroxylation/water retention capability, which is commonly associated with enhanced bioactivity.^33,34,53,55^ SEM images of the powders before and after immersion in SBF for up to 21 days are shown in Figure 9. Early stage bioactivity signs, such as incipient CaP precipitates, were randomly distributed across the powder surfaces. Because the samples were in powder form and the immersion duration was relatively short (i.e., 3 weeks), only thin CaP deposits were observed, rather than the cauliflower-like, fully developed apatite layers typically reported for pellets or scaffold-type samples after prolonged immersion (≥6 weeks).^51,54^

The proliferation of preosteoblasts in response to the microwave-assisted synthesized undoped β-TCP and Se-β-TCP powders was evaluated using the MTT assay, and the results are presented in Figure 10a. Cell proliferation increased over the 5 day culture period for all groups, indicating that both powders supported preosteoblast proliferation. On days 1 and 3, no statistically significant differences in the cell number were observed among the TCPS (tissue culture polystyrene) control, undoped β-TCP, and Se-β-TCP groups (*p* > 0.05). By day 5, cell proliferation continued to increase in all groups. Although no significant difference was observed between the undoped β-TCP and Se-β-TCP groups (*p* > 0.05), the Se-β-TCP group exhibited a slightly lower cell number than the TCPS control (*p* < 0.05), which may be attributed to the release of selenite ions into the culture medium. SEM images of preosteoblasts cultured for 3 days further supported these findings, showing a well-spread cell morphology in all groups with no observable adverse effect from selenite incorporation (Figure 10b–d).

**Figure 10:**
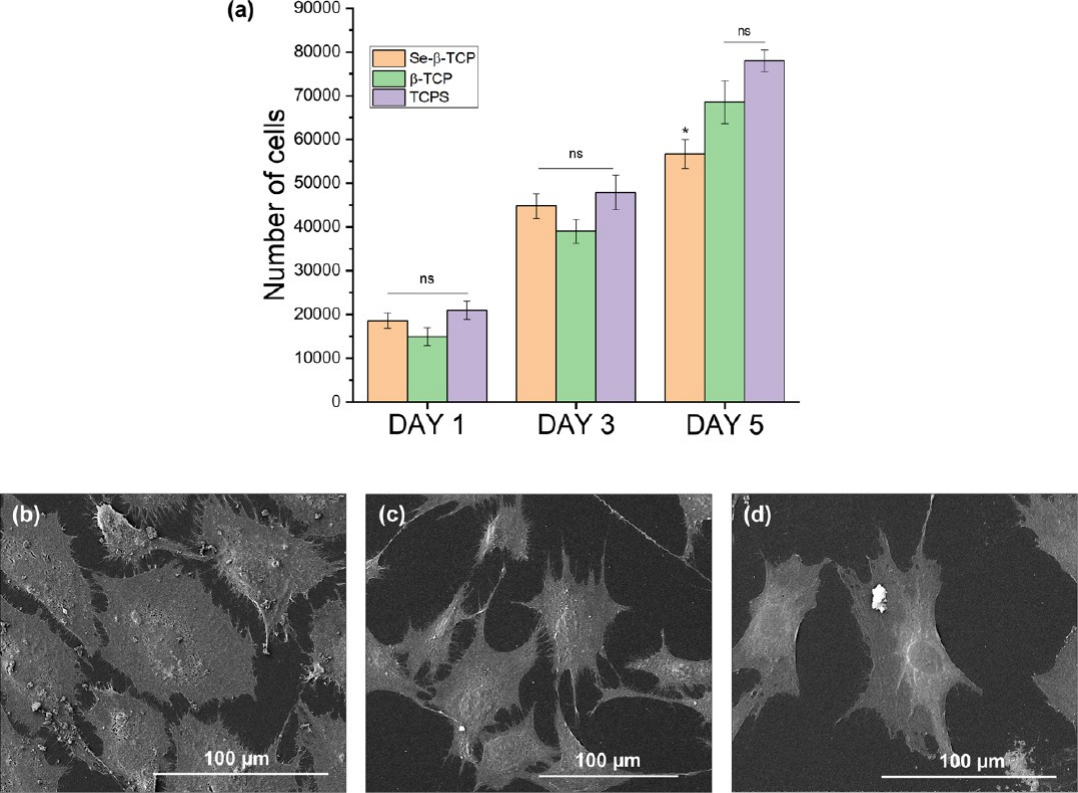
(a) The preosteoblast cell counts after culture with powder extracts for up to 5 days (ns: no significant difference; *p < 0.05). SEM images of preosteoblasts cultured for 3 days with (b) undoped β-TCP and (c) Se-β-TCP powders extracts and (d) TCPS, showing a well-spread cell morphology in all groups.

The antibacterial activity of the powders was evaluated by culturing *S. epidermidis*, *S. aureus*, and *E. coli* in 0.01 g/mL powder extracts, with powder-free samples (TCPS) serving as controls (Figure 11). After 24 h of incubation, the Se-β-TCP extracts resulted in a significant reduction in colony-forming units (CFUs) for all three bacterial strains compared to the TCPS control (Figure 11a–c), which was further corroborated by the corresponding agar plate images (Figure 11d–i). In contrast, β-TCP extracts did not reduce the CFU levels of *S. epidermidis* and *E. coli*, and only a slight decrease was observed for *S. aureus*. The antibacterial activity of the Se-β-TCP powders is therefore attributed to the release of SeO_3_^2–^ ions into the culture medium. These results are consistent with previous reports demonstrating that SeO_3_^2–^ ions inhibit the growth of both Gram-positive and Gram-negative bacteria.^8,38^ Nevertheless, the effect of the ion-doped powders is higher for Gram-negative bacteria, which can be attributed to the lower thickness and higher permeability of their cell wall structure, resulting in a less effective barrier against SeO_3_^2–^-doped β-TCP particles.^9,17,56^ The incorporation of the SeO_3_^2–^ ions into the microwave-assisted-synthesized undoped β-TCP structure supported preosteoblast proliferation and enhanced antibacterial performance.

**Figure 11:**
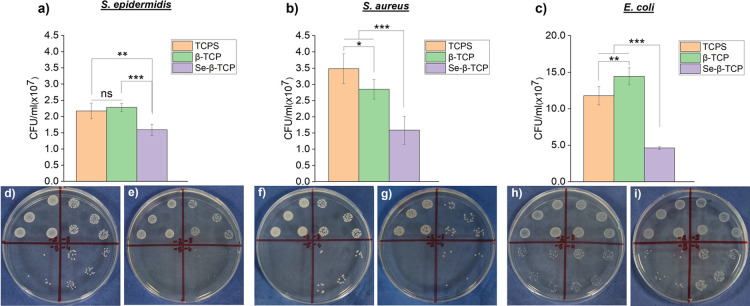
Number of (a) *S. epidermidis*, (b) *S. aureus*, and (c) *E. coli* colonies cultured with 0.01 g/mL powder extracts. Representative agar plate images of (d,e) *S. epidermidis*, (f,g) *S. aureus*, and (h,i) *E. coli* at various dilutions for (d,f,h) TCPS controls (no particles) and (e,g,i) 0.01 g/mL Se-β-TCP powder extracts.

Selenium (Se) is a well-established essential trace element in the human body. Selenium deficiency is associated with an increased risk of immune system dysfunction due to its critical role in the antioxidant defense system.^32^ Beyond its physiological importance, Se has been reported to exhibit anticancer activity, promote bone regeneration, and possess antimicrobial and antibacterial properties. It plays an essential role in osteogenic activity and is a key component of several proteins and enzymes involved in cellular function.^57–59^ The beneficial effects of Se-based nanoparticles in biomedical applications, particularly in wound healing and tissue regeneration, have been reported in several studies; however, further investigation is needed to fully elucidate their effects on cell viability and antibacterial activity in orthopedic applications. Unlike silver (Ag), which is widely used as an antibacterial agent but can exhibit cytotoxic effects at high concentrations or upon prolonged exposure,^57^ SeO_3_^2–^ incorporation has been shown to simultaneously enhance cell proliferation while inhibiting bacterial growth.^32,58,59^ Given that a substantial proportion of revision surgeries are associated with *S. aureus* infections, reducing the risk of implant-related bacterial colonization remains a critical clinical challenge.^58^ In this context, SeO_3_^2–^-doped β-TCP can be a promising approach. The antibacterial mechanism of Se is attributed to the catalytic oxidation of intracellular thiols and the generation of reactive oxygen species, such as singlet oxygen, which ultimately leads to bacterial cell death.^32,58^ Consistent with these reports, the present study demonstrated that SeO_3_^2–^ incorporation into microwave-assisted-synthesized β-TCP significantly reduced the number of bacterial colonies across all tested bacterial strains compared with undoped β-TCP, confirming the antibacterial potential of Se-doped β-TCP.

## 4 Conclusion

This study demonstrated a rapid, practical route to produce SeO_3_^2–^-doped β-TCP powders via microwave-assisted synthesis. Comprehensive characterization studies were performed to assess phase composition, molecular bonding, microstructure, particle morphology, and biological performance. The key findings are summarized as follows:

- Undoped β-TCP and SeO_3_^2–^-doped β-TCP powders were successfully synthesized via microwave-assisted processing, enabling the reaction between Ca and P sources to occur under 15 min microwave irradiation.
- XRD analyses revealed a reduction in the *a* and *c* lattice parameters and peak shifts toward higher diffraction angles, confirming the incorporation of SeO_3_^2–^ ions into the β-TCP crystal lattice.
- FT-IR results further supported SeO_3_^2–^ substitution, as evidenced by the characteristic Se–O vibration bands at 904–806 cm^–1^ and 816 cm^–1^.
- Both calcined undoped β-TCP and Se-β-TCP powders exhibited a rounded submicron particle morphology, indicating that selenite incorporation did not substantially alter particle shape.
- Se-β-TCP powders demonstrated antibacterial activity at a 0.01 g/mL extract concentration, attributed to the release of SeO_3_^2–^ ions, while still supporting preosteoblast proliferation over 5 days, highlighting the dual benefit of Se incorporation.

Overall, these results indicate that microwave-assisted synthesis is a promising strategy for developing multifunctional CaP-based powders. Future studies may explore dual- or multi-ion incorporation into β-TCP or other CaP phases using this approach to synergistically enhance various therapeutic functionalities, including degradation rate, osteogenic activity, and antimicrobial performance.
